# Adducin at the Neuromuscular Junction in Amyotrophic Lateral Sclerosis: Hanging on for Dear Life

**DOI:** 10.3389/fncel.2016.00011

**Published:** 2016-01-29

**Authors:** Charles Krieger, Simon Ji Hau Wang, Soo Hyun Yoo, Nicholas Harden

**Affiliations:** ^1^Department of Biomedical Physiology and Kinesiology, Simon Fraser UniversityBurnaby, BC, Canada; ^2^Department of Molecular Biology and Biochemistry, Simon Fraser UniversityBurnaby, BC, Canada

**Keywords:** amyotrophic lateral sclerosis, neuromuscular junction, adducin, Hu-li tai shao, Discs large, neural cell adhesion molecule

## Abstract

The neurological dysfunction in amyotrophic lateral sclerosis (ALS)/motor neurone disease (MND) is associated with defective nerve-muscle contacts early in the disease suggesting that perturbations of cell adhesion molecules (CAMs) linking the pre- and post-synaptic components of the neuromuscular junction (NMJ) are involved. To search for candidate proteins implicated in this degenerative process, researchers have studied the *Drosophila* larval NMJ and find that the cytoskeleton-associated protein, adducin, is ideally placed to regulate synaptic contacts. By controlling the levels of synaptic proteins, adducin can de-stabilize synaptic contacts. Interestingly, elevated levels of phosphorylated adducin have been reported in ALS patients and in a mouse model of the disease. Adducin is regulated by phosphorylation through protein kinase C (PKC), some isoforms of which exhibit Ca^2+^-dependence, raising the possibility that changes in intracellular Ca^2+^ might alter PKC activation and secondarily influence adducin phosphorylation. Furthermore, adducin has interactions with the alpha subunit of the Na^+^/K^+^-ATPase. Thus, the phosphorylation of adducin may secondarily influence synaptic stability at the NMJ and so influence pre- and post-synaptic integrity at the NMJ in ALS.

## The Neurodegenerative Disease Amyotrophic Lateral Sclerosis

The neurodegenerative disorder amyotrophic lateral sclerosis (ALS), also known as motor neurone disease (MND), is a motor system disease that causes progressive motoneuron loss in the spinal cord and brainstem leading to weakness and loss of muscle innervation (i.e., denervation), as well as the degeneration of descending motor tracts from the brain and subcortical structures resulting in spasticity (Eisen and Krieger, 2006; Su et al., 2014). Death typically occurs within 2–5 years of diagnosis, usually resulting from respiratory failure due to respiratory muscle weakness. At post-mortem, ALS patients have lost large numbers of spinal motoneurons, interneurons and other neuronal populations, but with considerable side-to-side asymmetry and variability at spinal and brain stem levels (Tsukagoshi et al., 1979; Swash et al., 1986). These findings are, by their very nature, end-stage effects and do not reflect the early stages of the disease. The large majority of ALS cases appear sporadically in the general population without evidence for an inherited gene mutation (“sporadic ALS”). However, genes associated with ALS susceptibility are being defined (Keller et al., 2014) and mutations in various genes are sometimes found in patients with sporadic ALS. In 5–10% of ALS patients, the disease has an autosomal dominant or, in rare cases, autosomal recessive inheritance, and is termed familial ALS (FALS; Andersen and Al-Chalabi, 2011). Gene mutations associated with FALS have been under intense scrutiny, and such mutations are currently identified in approximately 50% of FALS patients. Observed in the highest percentage of FALS patients are mutations in Cu/Zn superoxide dismutase 1 (SOD1) and chromosome 9 open reading frame 72 (C9ORF72; Vucic et al., 2014). However, mutations in TAR DNA-binding protein 43 (TDP-43, TARDBP), fused in sarcoma/translocated in liposarcoma (FUS/TLS), vesicle-associated membrane protein-associated protein B (VAPB) and other genes have been reported (Andersen and Al-Chalabi, 2011). The functional diversity of the proteins associated with these gene mutations (e.g., SOD1, a cytosolic anti-oxidant; VAPB, a synaptic membrane-associated protein; and others) makes it unlikely that a single disease-initiating event will be present, but rather that a number of unique triggering events will result in a common pathogenic process leading to muscle denervation. Mutations in genes for isoforms of the cytoskeletal protein adducin have not been reported in FALS patients nor have mutations been found in genes for proteins which interact with adducin. Rarely, missense mutations are found in genes for synaptic proteins such as VAPB, both in FALS and in sporadic ALS (Millecamps et al., 2010). Although the expression of mutant VAPB in *Drosophila melanogaster* is associated with neuromuscular junction (NMJ) defects such as increased numbers of synaptic boutons at the NMJ and decreased bouton size, as well as locomotor defects, VAPB has numerous effects in neurons and it is not possible to conclude that VAPB mutations produce a phenotype that results exclusively from synaptic dysfunction (Sanhueza et al., 2014).

There are some differences in the clinical features of FALS due to distinct mutations in comparison to sporadic ALS (Stewart et al., 2012). However, in the absence of a family history, it is virtually impossible to distinguish FALS patients from those with sporadic ALS, suggesting that sporadic and FALS are mechanistically similar and that insights into FALS will be relevant for an understanding of sporadic ALS. Although it is clear that specific gene mutations result in ALS, it is not clear how these mutations result in disease. In this review, we will focus on the evidence that ALS results from synaptic dysfunction at the NMJ as well as at other synapses. We recognize that there is also evidence for other explanations as causative for ALS. We will also highlight the importance of cytoskeletal proteins such as adducin as being a modulator for the synaptic dysfunction in ALS.

## Selective Vulnerability of Motoneurons in ALS

It is likely that ALS develops as a consequence of the selective vulnerability of motoneurons or the axonal pathways from the brain and brainstem that descend to motoneurons to undergo dysfunction and cell death. The descending motor tracts that innervate motoneurons include the corticospinal tract and other descending motor tracts from the brain and brainstem. The vulnerability of motoneurons is produced by the large size of these cells, their large caliber and long axons, the abundant dendritic projections, and the high metabolic cost of action potential generation in motoneurons (Le Masson et al., 2014). Additionally, there is possibly a toxic environment surrounding motoneurons, including the presence of microglia and astrocytes that might be responsible for non-cell autonomous toxicity (Julien, 2007; Ikiz et al., 2015). Furthermore, motoneurons exhibit an abundance of excitatory amino acid (EAA) receptors including N-methyl-D-aspartate (NMDA) receptors, and some of these receptor channels have calcium permeability. Stimulation of EAA and/or NMDA receptors can lead to excitotoxicity which is likely accompanied by raised intracellular Ca^2+^ concentrations ([Ca^2+^]_i_) in the cell body, but alternatively could occur in the post-synaptic region or pre-synaptic terminal. Motoneurons have lower levels of calcium buffering proteins, such as calbindin, than do other neuronal types, thus possibly enhancing the toxicity of elevated [Ca^2+^]_i_. Because of the vulnerability of the motoneuron to injury, previous hypotheses of neuronal dysfunction in ALS have focused on excitotoxicity, axonal dysfunction, or aberrant RNA processing in motoneurons. Elevations of [Ca^2+^]_i_ are capable of activating Ca^2+^-dependent protein kinases such as some isoforms of protein kinase C (PKC). One consequence of PKC activation is the increased phosphorylation of cytoskeletal proteins such as adducin (Krieger et al., 2003). As described below, adducin is present at the synapse both presynaptically and postsynaptically and exists in phosphorylated and non-phosphorylated forms. Non-phosphorylated adducin links actin and spectrin filaments and provides stability to the NMJ. When adducin is phosphorylated by PKC or other protein kinase adducin will translocate from the membrane, where it likely stabilizes the synapse, to the cytosol. This will result in a de-stabilization of the synapse which can be important for synaptic plasticity such as in learning but may reduce the ability of the NMJ to form strong connections between pre-synaptic and post-synaptic contacts. Thus indirectly, the elevations of [Ca^2+^]_i_ lead to destabilization of the synapse, especially at the NMJ, mediated in part through adducin. We recognize that this view is simplistic, as adducin effects are likely different at pre- and post-synaptic regions (Pielage et al., 2011; Wang et al., 2011, 2014) and that elevations in [Ca^2+^]_i_ will have many effects. Nonetheless, it is the purpose of this review to highlight the potential role played by cytoskeletal proteins in general and adducin in particular as mediators for some of the reactions of motoneurons to cell stress including impaired synaptic connectivity.

Also important is that the motoneuron is surrounded by non-neuronal cells such as astrocytes, microglia and terminal Schwann cells which when activated could result in motoneuron damage (Julien, 2007). Evidence for non-cell autonomous damage to motoneurons in the pathogenesis of ALS is strong given studies in transgenic mice that neuron-specific expression of mutant proteins generally does not result in much motoneuron loss whereas ubiquitous expression of mutant proteins will produce profound motoneuron dysfunction (Ilieva et al., 2009; Ikiz et al., 2015). Although not well studied, it is likely that the effects of adducin phosphorylation in non-neuronal cells will be different from the effects of phosphorylated adducin at the NMJ.

## ALS Initially Affects the Distal Motoneuron Axon and Neuromuscular Synapse

Attempts to delineate early changes in ALS, as opposed to those at end-stages, have focused on biopsy material from ALS patients and controls, as well as studies in widely used animal models of ALS such as transgenic rodents over-expressing human mutant SOD (mSOD), which manifest motoneuron degeneration similar to that seen in ALS patients (Turner and Talbot, 2008). Studies involving mSOD mice have shown that motoneuron loss is prominent but not complete such that approximately 50% of motoneurons are still evident even in animals in the terminal stages of disease (Parkhouse et al., 2008; Turner and Talbot, 2008). These observations demonstrate that motoneurons and their proximal axons are still present in the late stages of the disease, but that many of the axons are incapable of forming functional NMJs with skeletal muscle (i.e., denervation) leading to muscle weakness. Furthermore, these axons are often unable to re-establish stable contacts with muscle once denervation has occurred (i.e., impaired re-innervation). This view is supported by evidence that motoneurons in mSOD mice with advanced disease can still be identified using morphological or immunohistochemical techniques, yet, they will not manifest labeling with retrograde markers such as FluoroGold delivered to the muscle, which would normally be taken up by the presynaptic terminals and transported in the retrograde direction to the cell bodies (Parkhouse et al., 2008). This is presumably due to loss of axon integrity at either the proximal or distal axon (Kennel et al., 1996). The capacity for compensatory axonal sprouting is strikingly reduced in ALS, even when compared to another motor neuron disease such as poliomyelitis. Interestingly in patients who develop poliomyelitis and have considerable motoneuron loss, the few surviving motoneurons are able to re-innervate muscle and compensate for the reduction in motoneuron number even when the motoneuron losses are as extensive as in some patients with ALS (Trojan and Cashman, 2005). Several studies that examined biopsies obtained from patients with ALS early in the disease have confirmed that muscle denervation is extensive, and develops prior to significant motoneuron loss (Fischer et al., 2004). The impairment in the ability of motoneurons to re-innervate muscle may also be the case in normal aging, but to a much lesser extent (Valdez et al., 2012). The degenerative process of ALS may initially lead to impaired function of the distal axon (so called “axonopathy”), with relative preservation of the cell body and proximal axon, thus offering hope that if axonal function can be stabilized, then the axonal loss can be halted or even reversed and the disease progression can be attenuated. The role of axonal transport in neurodegenerative disease has been reviewed previously (De Vos et al., 2008).

The predominant features of ALS at the motoneuron, as distinct from axonal loss in the central nervous system (CNS), are weakness and fasciculations. Fasciculations are brief, spontaneous contractions of a limited number of muscle fibres which may arise from the spontaneous firing of motoneurons or their axons. Motor weakness is not exclusively found in ALS and can be present in many conditions due to impaired functioning of different regions of the motoneuron. For instance, some forms of neuropathy target the axon of the motoneuron (“axonopathy”). Some types of axonopathy have been referred to as a “dying back” process, where the longest and largest motor axons are targeted first, and the initial impairment affects the most distal portions of the axon (also called “distal axonopathy”), such as at the pre-synaptic terminal. Subsequently, more proximal portions of the axon become affected in the disorder. This view is based on neuropathological evaluation of some types of toxin-induced peripheral nerve damage (peripheral neuropathy) such as that following extensive alcohol consumption, where the fine intramuscular nerve branches of motoneurons may be affected initially while more proximal axons are spared. Presumably, the most distal aspects of the nerve are more susceptible to some toxins or to axonal “undernourishment”, or impaired axon transport associated with the actions of a toxin (Cavanagh, 1979). An additional clinical condition resulting in weakness that is likely due to impaired distal axon and NMJ function are some forms of critical illness polyneuropathy (Hermans and Van den Berghe, 2015). This evidence collectively supports the view that a loss of nerve-muscle contacts occurs early in the disease, which might represent the inability of presynaptic motoneuron terminals to re-innervate muscle at the NMJ (Moloney et al., 2014). However, it also should be kept in mind that transgenic mice with neuron-specific expression of mSOD do not develop disease with the same characteristics as with ubiquitous expression of mSOD, raising the likelihood that ALS is a disease not only of neurons, but also neurons and their non-neuronal neighbors (Julien, 2007; Ilieva et al., 2009; Meyer et al., 2014). These types of studies have not suggested that muscle cells were a cell type that is responsible for development of murine or human ALS (Miller et al., 2006).

Numerous factors are responsible for nerve-muscle interactions, both during development and at the mature synapse. The integrity of cell adhesion molecules (CAMs) that link the pre- and post-synaptic regions is undoubtedly relevant for the functionality of neuromuscular control. However, the link between structure and function at the NMJ is complex with numerous interactions between NMJ components and considerable functional redundancy for proteins at the NMJ (Koper et al., 2012). There are many evolutionarily conserved synaptic CAMs such as cadherins, integrins, neurexins and others. Physiological activation of the NMJ also has profound effects where pre- or post-synaptic cholinergic antagonism with botulinum toxin, or bungarotoxin, have similar effects to surgical denervation (Avila et al., 1989). It is also clear that perisynaptic Schwann cells (also called “terminal Schwann cells”) exert important roles in synapse re-organization at the NMJ in ALS (Arbour et al., 2015). As will be discussed below there is evidence that one of these CAMs such as a *Drosophila* orthologue of neural cell adhesion molecule (N-CAM) interact indirectly with synaptic adducin.

Mammalian motoneurons have different physical and firing characteristics that can be roughly grouped into distinct classes, such as fast-fatigable (FF) and slow (S; Burke, 1967; Heckman and Enoka, 2012). Interestingly, there are differences in the susceptibility of various synaptic subtypes to degeneration in ALS, with synapses of FF motoneurons being vulnerable to synaptic loss and synapses of slow motoneurons being relatively resistant (Frey et al., 2000; Pun et al., 2006; Hegedus et al., 2008). In addition, with disease there may be an activity-dependent conversion of motoneuron types towards a slow phenotype (Frey et al., 2000). The progressive weakening of specific synapses with disease suggests synapse-specific mechanisms as being at least partially responsible for selective synaptic loss. This view is supported to some degree by the observation that the WldS gene, which protects against axonal injury, modestly prolongs survival in the mSOD mouse and other murine models of ALS (Ferri et al., 2003; Fischer et al., 2005). Synaptic terminals that are less susceptible to denervation also demonstrate more ability to generate stimulus-induced synaptic sprouting, which could arise from differences in the regulation of the actin cytoskeleton of the different synaptic types (Laux et al., 2000).

One additional piece of circumstantial evidence for the presence of distal axon or synaptic terminal dysfunction in ALS is the finding of decremental responses in muscle to repetitive nerve stimulation both clinically in ALS patients and in models of ALS (Kennel et al., 1996; Iwanami et al., 2011; Piotrkiewicz and Hausmanowa-Petrusewicz, 2013). That is, when motoneuron axons are stimulated repetitively, there is a reduction in the amplitude of the evoked response in muscle suggesting an impairment of neuromuscular transmission at the NMJ. ALS also includes involvement of CNS structures and it has not yet been determined whether involvement of the motoneuron precedes dysfunction in descending axons, or if the two processes occur concurrently, or in the reverse order (Dengler, 2011).

## Mitochondrial Dysfunction in ALS

Mitochondrial dysfunction has been linked with many neurodegenerative conditions, including ALS (Schon and Przedborski, 2011; Cozzolino et al., 2015). However, it is not clear how mitochondrial dysfunction arises in ALS and whether the impairment is an initiating feature of the disease, or a consequence of the motoneuron dysregulation that arises from other causes. Impaired mitochondrial function likely leads to defective bioenergetics, but could also produce defective mitochondrial trafficking, impaired mitochondrial fusion and fission, as well as altered mitochondrial quality control (Schon and Przedborski, 2011). For instance, mitochondrial disruption has been reported to contribute to SOD1-mediated motoneuron degeneration (Jaiswal and Keller, 2009). Motoneurons are large, actively firing cells having very demanding ATP-dependence to maintain the resting membrane potential, neurotransmitter release and other functions. Of relevance to this review are observations that the cytoskeletal protein adducin is associated with and directly interacts with the α-subunit of the Na^+^/K^+^-ATPase (Ferrandi et al., 1999; Torielli et al., 2008). Mutations in α-adducin are associated with hypertension in rats and humans which is presumably based on aberrant interactions between the mutant α-adducin and Na^+^/K^+^-ATPase (Torielli et al., 2008). Although it is beyond the scope of this review to discuss mitochondrial function in ALS, it is relevant that a major energy consumer in the neuron is the Na^+^/K^+^-ATPase which is essential for providing energy for neuronal repolarization following an action potential. Recent modelling studies of ATP utilization in model neurons have suggested that the amount of ATP required for ion homeostasis is closely matched by energy production (Le Masson et al., 2014). Under conditions where motoneurons produce high frequency action potential firing, especially in fast fatiguable motoneurons, reductions in steady state ATP levels can occur. Modelling studies of action potential firing in excess of 20 Hz suggest that motoneurons cannot support continued Na^+^/K^+^-ATPase activity and neurons will depolarize (Le Masson et al., 2014). This also might lead to aberrant action potential firing, such as is seen clinically in the appearance of “fasciculations”, the spontaneous discharges of motor units that may be related to abnormal motoneuron discharge (de Carvalho et al., 2014). Furthermore, modelling studies also suggest that the energetic cost to support the vulnerable, “FF” motoneurons is higher than for the “S” motoneurons providing a possible explanation for the differential vulnerability of the two motoneuron subpopulations as is seen in ALS (Le Masson et al., 2014). Fasciculations arise from spontaneous motoneuron firing at the cell body, axon or other sites (de Carvalho et al., 2014).

## Could Molecules Mediating Cell Adhesion be Relevant for the Pathogenesis of ALS?

One approach to determine which molecules could be relevant for the pathogenesis of ALS would be to compare the biochemical profiles of synapses that are retained in ALS with those that are lost. This can be attempted by comparing NMJs from biopsy material from the limb muscles that are typically involved in ALS, with NMJs in extraocular muscles, which usually are not involved in ALS. One such study has claimed that significant reductions in the immunoreactivity of the extracellular matrix protein, α4 laminin, are seen in the NMJs of limb muscles in ALS patients, but are not as affected in the NMJs of extraocular muscles (Liu et al., 2011). It is possible that these immunohistochemical findings are related to the disruption of the NMJ, rather than being causative, but the results suggest that the integrity of the basement membrane surrounding the muscle fibre may be important for synapse stability. Interestingly, removal of Laminin A in *Drosophila* embryos causes a reduction in NMJ adhesion, and it has been proposed that the basement membrane contributes to adhesion of the motoneuron terminal to the muscle (Koper et al., 2012).

A potentially relevant CAM for ALS is the N-CAM and its homologue in the fly, Fasciclin 2 (Fas2). Mice lacking N-CAM in presynaptic terminals have impaired muscle re-innervation following denervation, emphasizing the important role played by N-CAM for re-innervation (Chipman et al., 2014a). It is also well established that at the simpler *Drosophila* NMJ, Fas2 is necessary for synaptic stabilization and plasticity (Schuster et al., 1996a,b). Furthermore, the proportion of denervated muscle fibres in transgenic mice overexpressing mSOD as identified using N-CAM immunoreactivity correlates well with the rate of decline of muscle contractile force in these mice (Gordon et al., 2009). When pluripotent stem cells are directed to differentiate into motoneurons, motoneuron stem cells lacking N-CAM have well defined structural and functional deficits at the NMJ (Chipman et al., 2014b). These deficits may be less pronounced than when N-CAM is lost from both pre- and post-synaptic sites. The mechanisms underlying the actions of N-CAM and Fas2 in modulating re-innervation at the NMJ are under investigation. However, it would seem important to study proteins that could have an impact on these CAMs and that these molecules deserve further scrutiny in ALS. In the following section we focus on a cytoskeletal protein that interacts with many other proteins at the NMJ and is likely relevant for understanding the pathophysiology of ALS.

## Adducin at the NMJ

### The Distal Axon and Synapse are Regulated by the Actin Cytoskeleton

**Figure 1:**
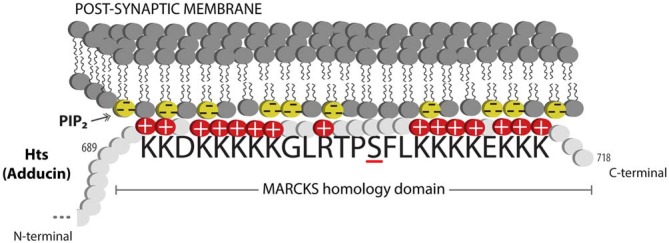
**Adducin hangs on to the membrane.** Schematic diagram showing the MARCKS domain of Adducin/Hts (amino acid sequence shown) acting to sequester PIP2 (shown as yellow lipids) in the plasma membrane. Protein kinase C (PKC) phosphorylation of the serine (underlined) in the basic effector domain of MARCKS reduces the electrostatic interaction with the membrane and causes translocation of adducin/Hts from the plasma membrane to the cytoplasm (see Figure 2).

**Figure 2:**
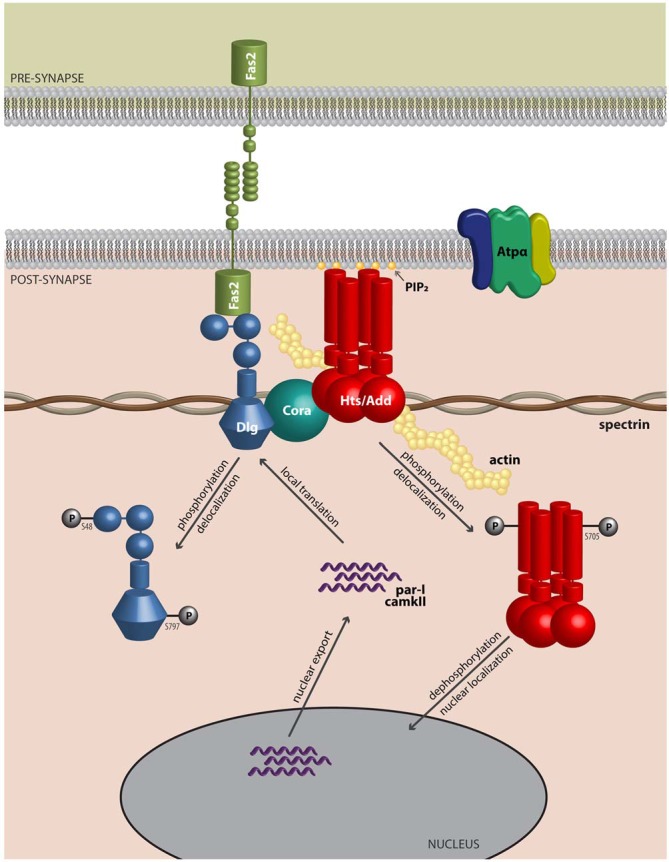
**Adducin hangs on to the cytoskeleton where it modulates synaptic structure.** Model of Adducin/Hts and interacting proteins at the neuromuscular junction (NMJ). Model shows a lateral view of an NMJ with presynaptic membrane shown above. Post-synaptically, Adducin/Hts (“Hts/Add”) is localized to actin-spectrin junctions and is found associated with Dlg, possibly through binding to Coracle (Cora). Homophilic adhesion between Fas2 molecules links the pre- and post-synaptic membranes, with the intracellular domain of Fas2 anchored to Dlg. Also associated with Hts is the α-subunit of the Na^+^/K^+^-ATPase. Based on studies of mammalian adducin, we speculate that when phosphorylated, Hts translocates from the membrane and its subsequent dephosphorylation may enable Hts access to the nucleus. In the nucleus, Hts is involved in export of transcripts for the kinases PAR-1 and CaMKII. PAR-1 and CaMKII subsequently phosphorylate the scaffolding molecule Dlg, which delocalizes from the NMJ, leading to reduced adhesion between nerve and muscle. In this model Hts monitors the status of the NMJ and can promote synaptic plasticity by shuttling to the nucleus.

Actin and spectrin are cytoskeletal proteins whose interactions are critical for the morphology of the presynaptic terminal and the post-synaptic region. The association of these proteins is relatively weak, and spectrin requires accessory proteins to assemble and stabilize spectrin-actin interactions, which include the cytoskeletal accessory proteins adducin and protein 4.1 (Bennett and Baines, 2001). These interactions permit the cytoskeleton to provide several functions that have been clarified through study of the erythrocyte membrane: they allow structural support through the lattice-like arrangement of the fibrils of filamentous actin and spectrin; they provide a platform on which many accessory proteins can bind; and through the variation in the lattice sizes of actin and spectrin, they can likely modulate the structural strength of local regions of the membrane. Adducin is a tetramer composed of α, β and γ subunits, where interactions with actin and spectrin are mediated by the carboxy-terminal region of adducin that has homology to a myristoylated alanine-rich C-kinase substrate (MARCKS)-related domain (Matsuoka et al., 2000). This highly basic, charged MARCKS domain likely permits adducin to associate (i.e., “hang on”) to the cell membrane, and sequester highly charged phosphoinositides (PIs) such as PI(4,5)P_2_ (Denisov et al., 1998; Figure 1). Adducin also associates with the sides and fast-growing ends of actin filaments, and exhibits high affinity for actin-spectrin complexes (Matsuoka et al., 2000; Figure 2). In this sense, adducin “hangs on” to the cytoskeleton and thus serves as a linker between the cell membrane and the cytoskeleton. The MARCKS region contains a phosphorylation site that is modulated by Ca^2+^/calmodulin and PKC, where the phosphorylation of adducin inhibits the stabilization of actin-spectrin complexes (Matsuoka et al., 2000; Figure 2). Interestingly, mammalian adducin and its invertebrate homologues have a number of defined synaptic protein-protein interactions including Na^+^/K^+^-ATPase, Discs large (Dlg) and Golden Goal (Gogo; Ohler et al., 2011; Wang et al., 2011, 2014; Gallardo et al., 2014; Figure 2).

### A Role for Adducin and Phosphorylated Adducin in ALS

Adducin is of interest in ALS as there are high levels of phosphorylated adducin (phospho-adducin) protein in spinal cord tissue from patients who died with ALS compared to individuals who died without neurological disease (Hu et al., 2003). Furthermore, immunocytochemistry of murine spinal cord tissue demonstrated that phospho-adducin immunoreactivity was significantly higher in mSOD spinal cord tissue compared to control tissue (Shan et al., 2005). Recently, both of these observations have been confirmed and the association between adducin and ALS extended (Gallardo et al., 2014). It is possible that excessive levels of phosphorylated adducin would create instability at the NMJ and knockdown of α-adducin by RNA interference (RNAi) in mSOD over-expressing mice results in greater motoneuron survival than in the absence of RNAi. The effects of elevations in phosphorylated adducin are likely not restricted to motoneurons as knockdown of α-adducin in the astrocyte population of co-cultured wild-type motoneurons with astrocytes over-expressing mSOD resulted in less motoneuron death. Gallardo et al. (2014) suggest that α-adducin mediates motoneuron degeneration in mSOD mice and potentially in human ALS as well. Evidence supporting involvement of adducin in ALS is shown in Table 1. Both we and Gallardo et al. (2014) have expressed the view that the effects of adducin in ALS are not mediated entirely by adducin, but are likely dependent on interactions between adducin and other proteins. Gallardo et al. (2014) have focused on interactions between α-adducin and the Na^+^/K^+^-ATPase, a protein that has been well-established to associate with adducin where this interaction has been of considerable interest with regard to the role of adducin polymorphisms in essential hypertension (i.e., high blood pressure). To our knowledge, the possible relevance of polymorphisms in α-adducin genes for the development of ALS has not been evaluated.

**Table 1:** **Adducin in nervous system function and dysfunction**.

**(A) Evidence for involvement of adducin in nervous system function.**
Adducin is found in the pre- and post-synapse of the NMJ in the fruit fly, and regulates synaptic plasticity.	Pielage et al. (2011) and Wang et al. (2011)
Mice lacking β-adducin exhibit impaired synaptic plasticity and learning.	Porro et al. (2010)
β-adducin is required for stable synapses and learning in “environmentally-enriched” environments.	Bednarek and Caroni (2011)
Memory retention in *C. elegans* is regulated by adducin.	Vukojevic et al. (2012)
**(B) Evidence that neurological disease is associated with altered adducin.**
Mutations in γ-adducin are associated with inherited cerebral palsy.	Kruer et al. (2013)
Chorein, a protein responsible for a movement disorder in humans, interacts with β-adducin.	Shiokawa et al. (2013)
**(C) Evidence that misregulated adducin is associated with ALS and models of ALS.**
Elevated levels of phospho-adducin in patients with ALS at autopsy.	Hu et al. (2003)
Elevated levels of phospho-adducin in a murine model of ALS.	Shan et al. (2005)
Knockdown of α-adducin in a murine model of ALS results in longer animal survival.	Gallardo et al. (2014)
Knockdown of α-adducin in astrocytes from murine ALS co-cultured with motoneurons results in motoneuron survival.	Gallardo et al. (2014)
The RNA-DNA binding protein, TDP-43, regulates β-adducin transcript stability.	Costessi et al. (2014)

The work of Gallardo et al. (2014) indicates that a gain of adducin function may have a causative role in ALS, especially in astrocytes, whereas a recent study from Costessi et al. (2014) proposes that a loss of adducin function may be involved. TDP-43 is an RNA-binding protein found to be a major component of pathological cytoplasmic inclusions in ALS and as such, perturbed TDP-43 function may be linked to neurodegeneration. Costessi et al. (2014) demonstrated that TDP-43 regulates adducin 2 gene expression by promoting adducin 2 mRNA stability, and suggest that loss of adducin function through disruption of TDP-43 activity may contribute to ALS.

These various studies indicate that misregulation of adducin activity through hyper-phosphorylation or changes in gene expression may be a contributing factor in the etiology of ALS. An alternative view could be that as many proteins are hyper-phosphorylated in ALS, adducin phosphorylation may be just one of many hyper-phosphorylated proteins in the disease (Krieger et al., 2003), and of little direct relevance. However, although not a “primary cause” for ALS, it is also possible that the hyper-phosphorylation or other misregulation of adducin is responsible for some of the particularly malicious aspects of ALS, namely the inability of denervated presynaptic terminals to re-innervate muscle and sustain life.

### Two Functions of Adducin at the *Drosophila* NMJ: Stabilizing the Presynaptic Terminal and Regulating Adhesion in the Post-Synaptic Region

**Figure 3:**
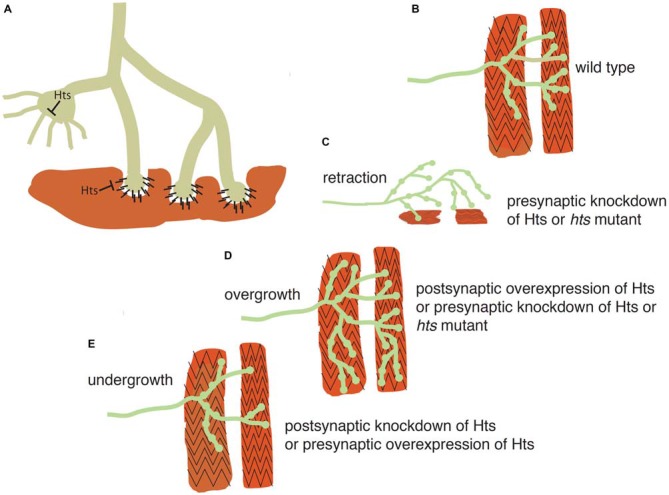
**Adducin maintains presynaptic cytoskeletal organization, and also regulates contacts between the presynaptic terminal and muscle. (A)** Schematic model showing action of adducin (“Hts”) in stabilizing actin-spectrin interactions in the motoneuron axon and presynaptic region (green). The actin-capping function of Hts may restrict actin-based outgrowths that participate in synapse elaboration. Postsynaptically (red), Hts can inhibit adhesion between muscle and bouton. **(B)** Schematic of *Drosophila* wild-type larval body wall muscles (red) being contacted by a motoneuron (green). **(C,D)** Knockdown of presynaptic adducin/Hts causes both overgrowth of the synaptic arbor and its retraction from the muscle. The same phenotype is seen in Hts mutant larvae. Overgrowth of the synaptic arbor is also seen with postsynaptic overexpression of Hts, probably as a result of reducing adhesion between nerve and muscle. **(E)** Post-synaptic loss of Hts prevents synaptic growth, possibly due to increased adhesion between nerve and muscle. The same phenotype is seen with presynaptic overexpression of Hts, probably due to inhibition of actin-based outgrowths.

Because of the strengths of *Drosophila* genetics, researchers have turned to the *Drosophila* larval NMJ for evaluation of adducin/Hts function in the nervous system. Presynaptically, spectrin-actin lattices are important for establishing a platform for regulating neurotransmitter release. Postsynaptically, these lattices are also present where they are responsible for the spacing and efficacy of the NMJ (Pielage et al., 2006). Two groups have shown that adducin/Hts is present in both the pre- and post-synaptic regions and that perturbations of adducin/Hts levels can influence the extent of synaptic branching of the NMJ (Pielage et al., 2011; Wang et al., 2011; Figure 3).

Adducin/Hts acts to maintain the presynaptic cytoskeletal organization, as well as to preserve contacts between the presynaptic terminal and muscle, as shown diagrammatically in Figure 3. Pielage et al. (2011) focused on the role of pre-synaptic adducin/Hts, where they provide evidence that it regulates synaptic structure through its role as an actin-capping protein (Figures 3A,B). Of particular interest is their finding that loss of pre-synaptic adducin/Hts causes both overgrowth of the synaptic arbor and its retraction from the muscle, a phenotype particularly relevant to ALS (Figures 3C,D). By contrast, post-synaptic adducin/Hts has a distinct role from pre-synaptic adducin/Hts, which acts as an inhibitor of adhesion between nerve and muscle. The ability to break adhesion and then re-form contacts between nerve and muscle is important for growth of the synapse during development (Schuster et al., 1996a), and the loss of post-synaptic Hts prevents synaptic growth, possibly due to increased adhesion between nerve and muscle (Figure 3E). Conversely, post-synaptic overexpression of Hts may promote synaptic overgrowth by reducing adhesion between nerve and muscle (Figure 3D).

Over-expression of adducin/Hts in the muscle results in delocalization of the key postsynaptic protein, Dlg. Co-immunoprecipitation and proximity ligation assay experiments demonstrate that adducin/Hts is present in a complex with Dlg at the NMJ (Wang et al., 2011, 2014, 2015). These results suggest that the synaptic function of adducin/Hts may be mediated, at least in part, through interactions with Dlg. But how does Hts regulate Dlg postsynaptic targeting? A relevant finding is that adducin/Hts over-expression leads to increases in muscle immunoreactivity against PAR-1 and CaMKII, two protein kinases known to phosphorylate Dlg and disrupt its localization at the post-synaptic membrane (Koh et al., 1999; Zhang et al., 2007; Wang et al., 2011). Thus, it appears that adducin/Hts regulates synaptic size by controlling Dlg localization at the postsynaptic membrane via PAR-1 and CaMKII-mediated phosphorylation. Dlg interacts with Fas2, which, as mentioned above, is the homolog of mammalian N-CAM (Thomas et al., 1997). Previous studies indicate that Dlg and Fas2 regulate synaptic plasticity and stabilization, and the *Drosophila* NMJ may prove ideal in addressing regulation of synaptic adhesion by adducin/Hts.

### Adducin is Present in Synapses of the Mammalian CNS

This review has focused on the action of adducin in relation to ALS through its action at the motoneuron and NMJ. However, ALS also affects the CNS, and adducin and phospho-adducin are widely distributed throughout the mammalian nervous system, especially in axons and presynaptic nerve terminals, regions that are likely relevant for the initiation of neurodegeneration in ALS (Seidel et al., 1995; Xu et al., 2013). Of interest is the observation that β-adducin knock-out mice have defects in learning, as well as long-term potentiation and long-term depression (Porro et al., 2010). β-adducin may be required specifically for the establishment of synapses in the CNS under conditions of an “enriched environment”, an environment where animals are exposed to more sensory stimuli than normal which results in augmented synaptogenesis and improved learning ability (Bednarek and Caroni, 2011). These effects of β-adducin in learning are thought to be due to adducin augmenting the outgrowth of one type of synaptic input to inhibitory interneurons in the hippocampus (Ruediger et al., 2011). It is tantalizing to speculate that the specific effect of adducin in synaptic circuitry might occur through an interaction with Dlg. A mammalian homologue of Dlg is PSD-95, a post-synaptic protein found in excitatory, but not at inhibitory synapses. Thus, modulation of adducin at excitatory and inhibitory synapses would occur differently, as it is likely that adducin would also be found at inhibitory synapses. Adducin is also found in cerebellar Purkinje cells where it associates with an enzyme that generates inositol pyrophosphates (inositol hexakisphosphate kinase-3; IP6K3) and IP6K3 knockout mice have impaired motor learning and coordination, possibly related to impaired disposition of adducin (Fu et al., 2015).

Interestingly, the nematode homologue of adducin, Add-1, has also been drawn into the debate about whether the act of forgetting memories is an active or passive process. Studies in *C. elegans* have suggested that the long term retention of learned material in this animal is facilitated by Add-1 and inhibited by single stranded RNAs of the mushashi (msi-1) family (Hadziselimovic et al., 2014). These data underscore that adducin has been involved in synaptic architecture for a long time from an evolutionary point of view and is central to synaptic function even in simple organisms.

Circumstantial evidence for the importance of adducin/Hts in CNS function is also provided by recent studies showing that mutations in γ-adducin are associated with familial cerebral palsy (Kruer et al., 2013). Additionally, adducin has been reported to be a binding partner for chorein, a protein believed critical for development of the movement disorder chorea-acanthosis (Shiokawa et al., 2013).

### Adducin: A Reporter of Synaptic Activity at the Nucleus and a Regulator of Transcription at the Synapse?

Although this review has focused on the role of adducin/Hts at the NMJ, there are studies that indicate nuclear functions for adducin that might be relevant to its roles at the synapse. α-adducin contains nuclear localization and export signals, and has been shown to translocate to the nucleus in cultured epithelial cells upon loss of cell-cell adhesions where it participates in mitotic spindle assembly (Chen et al., 2011; Chan et al., 2014). Interestingly, the nuclear localization and export signals are conserved in Hts, and Hts can localize to the muscle nucleus where it may regulate the export of transcripts that encode for the PAR-1 and CaMKII kinases, but not for transcripts that encode for Dlg (Wang et al., 2014; Figure 2). These various results raise the interesting possibility that Hts/adducin may shuttle between the NMJ and the nucleus in its regulation of the synapse. There is considerable evidence that protein trafficking occurs from the synapse to the nucleus especially with regard to activity-dependent gene expression (Kaushik et al., 2014). Furthermore, there is increasing recognition of misregulation of localized translation at the synapse in ALS, and adducin may also be involved in this process (Sephton and Yu, 2015).

## Conclusions

There are many independent primary causes for developing ALS. Mutations have been found in many genes in FALS involved in disparate processes including mutations in SOD1, TDP-43, C9ORF72 and other genes. Furthermore, the sporadic (non-familial) form of ALS has been associated with other risk factors and it is likely that ALS is a heterogeneous disease in which a variety of distinct initiating factors leads to common clinical manifestations. Current evidence suggests that the motoneuron loss in murine models of ALS are non-cell autonomous and require interactions between motoneurons, corticospinal tract neurons and their non-neuronal neighbors such as microglia, astrocytes and/or terminal Schwann cells. A particularly malicious aspect to ALS, and one that leads to the rapid decline of affected patients, is the inability of synaptic terminals to innervate their targets, particularly muscle, and especially respiratory muscle. This failure of synaptic connectivity might hinge on the ability of adducin to act with interacting proteins to permit strengthening of weakened synapses and allow the NMJ to “hang on” simultaneously to both terminal axons and muscle. The possible involvement of adducin and phospho-adducin in ALS suggests strategies for treatment. For instance, if the phosphorylation of adducin leads to some of the neurodegenerative changes, one approach would be to attempt to dephosphorylate adducin, or to inhibit the phosphorylation of adducin which could lead to a strengthening of the pre- and post-synaptic contacts, but which might exhibit less plasticity. This effect might be achieved pharmacologically by interfering with protein phosphorylation using compounds that block protein phospho-sites with sugar residues (Shan et al., 2012).

## Author Contributions

CK wrote the review. CK and NH were jointly responsible for writing and editing the manuscript and coordinating the figure preparation. SJHW and SHY were responsible for carrying out experiments relevant for this review, writing of the manuscript and for figure preparation.

## Conflict of Interest Statement

The authors declare that the research was conducted in the absence of any commercial or financial relationships that could be construed as a potential conflict of interest.
